# Challenges in Translating Academic Research into Therapeutic Advancement

**DOI:** 10.3389/fneur.2013.00123

**Published:** 2013-09-03

**Authors:** Gabriela Matos, Sergio Tufik, Monica Levy Andersen

**Affiliations:** ^1^Departamento de Psicobiologia, Universidade Federal de São Paulo, São Paulo, Brazil

**Keywords:** academic research, pharmaceutical companies, challenges, reproducibility of results, break of paradigms

In the last years, many pharmaceutical companies have closed their doors of research and development department, which in turn, resulted in the transference of commercial investments to universities. The commercial trend will become academic research more important than ever. Nowadays, the collaboration between the scientific community and companies becomes a reality. In this sense, how many academic studies have been published in compliance with international guidelines required for regulatory agencies? Unfortunately, few of them. The reproducibility of academic studies is a challenge that affects directly the success of a new clinical trial conducted by pharmaceutical industries.

This scenario was recognized by academic scientists and a platform for independent validation was created for articles already published^1^. Indeed, actions such as new editorial measures have just introduced in order to reduce the irreproducibility of scientific studies^2^. In this context, it is required the transparency of methods section, which will benefit the evaluation of the Reviewers and the reproduction of the study as well. For instance, it is mandatory the detailed description of reagents, inclusion and exclusion criteria, blinding, biological replicates, and accession codes for genetic material in animal studies.

However, there are other factors inherent in any academic researcher that are often ignored and undoubtedly corroborate to pitfalls in scientific results. We would like to expose here some suggestions for Academy to improve their work and increases the probability in translate their researches into commercial setting.

First of all, the experimental design of any project should be conducted considering the ultimate target of every study: the human being. Unreal situations or vague advances that do not reflect reliable patterns of “improvement” could be re-designed to be smart and feasible studies. For example, the common phrases in academic research such as “positive effects” or “preclinical and clinical evidence” become “efficient, safe, and commercially viable” inside industry (1). Reproducibility of results from the laboratory bench is a challenge. For instance, in oncology research, only 11% of targets identified in preclinical studies were verified as reliable for development in early stage industry studies, and up to two-thirds of projects based on published literature end up terminated due to lack of reproducibility (1, 2). This occurrence is also very common in neuroscience research. Poor reproducibility was the first point addressed in the guidelines for preclinical research in epilepsy (3) and demonstrated by the “Facilities of Research Excellence-Spinal Cord Injury” project (4–6). At the same time, the cost of development for a new anti-seizure therapy is around one billion dollars for industry, and in the last decades few therapies were successfully applied in patients with uncontrolled seizures (3). In view of these facts, it is hard for the pharmaceutical industry to apply the knowledge emerging from the majority of academic studies. Although it is very exciting that a substance “X” improved the neurological tissue in the brain; can this substance modulate the main symptom of a specific neurological disease? Can this substance reliably induce a significant effect in different strains of cells, *in vivo* models, and humans? We agree that this “ideal” approach to research is hard to establish mainly in laboratories with limited grants for extensive studies. In this context, collaborations between different departments, universities, and research fields could be an excellent alternative. Furthermore, is a unique opportunity to experience other point-of-views and researches fields, which could in turn, facilitate the development of new innovative and audacious ideas. The multidisciplinary staff is absolutely required for projects that result in robust data and significant advances, and not only an article that will be stored in a drawer. An interesting example of collaboration comes from private side: in 2012, the 10 biggest pharmaceutical companies joined and launched the TransCelerate Biopharma Inc, a non-profit organization with a common goal: to accelerate the medication development.

The second point that deserves discussion is the beliefs and paradigms of academic scientists. It is hard for academic researchers to accept that extensive and difficult work performed in their laboratories may result in no significant effect or even findings that oppose the current literature, both of which are extremely difficult to publish. This position is understandable if it takes into account the time spent on a project. In many cases, a single researcher is responsible for the entire experimental design. Opposite effects or insignificance are two of the greatest fears of any academic scientist, all of whom need to publish more than ever. However, if experiments with negative results meet all the necessary quality criteria (i.e., replication of experiments), unexpected or contradictory data should be published and well accepted by the editors, reviewers, and general scientific community (2). The break of paradigms regarding negative results should be priority in the academic research. For instance, phosphodiesterase type-5 inhibitor (PDEI-5) is a first-line oral treatment for erectile dysfunction in men (7, 8). The clear beneficial effects were extensively described in the literature, and after 10 years of analysis of adverse effects, PDEI-5 is considered safe with minimum adverse effects in men (9, 10). However, case reports and data from animal models indicated that, in particular cases, PDEI-5 should be prescribed with caution, as it may play a role in seizure susceptibility (11). This is an excellent example of why journals should accept papers with robust data commonly different from those published in the literature. In addition, the lack of negative results represents a direct problem for clinical translation of new compounds, medications, and devices. Publish negative data can avoid that other colleagues and pharmaceutical industries waste money and time in the conduction of non-significant experiments (12).

On the other hand, academic professionals are constantly pressured to publish, which in turn results in grants, prestige, and career advancement (13–15). In the majority of scientific journals, it is mandatory to declare any conflict of interest. According to The American Academy of Neurology and its affiliated organizations (AAN), those who have any relationship with industry (medical devices, pharmaceutical, or another commercial product), and receive benefits from this relationship, could be influenced by financial conflicts of interest (16). This type of financial conflict is very common among physicians and is the subject of many reviews and international guidelines (17). However, the “publish or perish” culture of academia is not yet acknowledged to represent a similar conflict of interest. We recognize that desire for success is an inherent feeling in any scientist; however, the quality and reliability of data must be the priority for any academic competition. Fortunately, the first step of adequate individual scientific performance evaluation was made: according the recommendations of the San Francisco Declaration on Research Assessment the journal impact factor will not take into account in the *curricula vitae* (18).

A meeting composed by academic and business leaders discussed the fragile relationship between Academy and industry^3^. They recognized the urgency to strengthen this link. This is the third and perhaps the major obstacle inside the walls of university: to break the prejudice of academic researches into work together with industry. However, it is important for researches to known that the better institutions around the world received important investments from the giant pharmaceutical industries, which joined with other companies mainly in clinical trials. The development of “collective” science is a trend inside industry (19) and Academy could gain with new business posture. Investments in infrastructure, staff, and supplies can be the major advantages for this collaboration. There are global programs from pharmaceutical industries available for all researches which would like to test their products.

As described above, the difficulties in translating academic findings to industry successes may be influenced by problems inherent in the current academic system. It is important for all of us to remember, regardless of ideology and goal of any science professional, academic, or industry, that we all have the same objective: the progress of science.

## Footnotes

^1^https://www.scienceexchange.com/reproducibility

^2^http://www.nature.com/news/announcement-reducing-our-irreproducibility-1.12852

^3^http://www.icsu.org/publications/cfrs/scientific-relations-between-academia-and-industry-building-on-a-new-era-of-interactions-for-the-benefit-of-society
